# Seizures in febrile children with SARS-CoV-2 infection: clinical features, short-term follow-up

**DOI:** 10.1186/s12887-024-04691-5

**Published:** 2024-04-17

**Authors:** Jipeng Jiang, Zhengsong Shen, Jie Cao

**Affiliations:** 1 Department of General Medicine, Nation Clinical Research Center for Child Health and Disorders, Ministry of Education Key Labiratory of Child Development and Disorders, Childrens Hospital of Chongqing Medical University, No.136 Zhongshan 2nd Road, Yuzhong District, Chongqing, China; 2 China International Science and Technology Cooperation base of Child development and Critical Disorders, No.136 Zhongshan 2nd Road, Yuzhong District, Chongqing, China; 3 Chongqing Key Laboratory of Pediatrics, No.136 Zhongshan 2nd Road, Yuzhong District, Chongqing, China

**Keywords:** COVID-19, Seizures, Follow-up, Acute necrotizing encephalopathies

## Abstract

**Background:**

As the Omicron variant of the Severe Acute Respiratory Syndrome Coronavirus 2 (SARS-CoV-2) emerges, the neurological manifestations correlated with this epidemic have garnered increasing attention. This study was primarily intended to compare seizures in febrile children with and without SARS-CoV-2 infection and to conduct short-term follow-up of the SARS-CoV-2-infected patients.

**Methods:**

Retrospective analysis of patients admitted to the Children’s Hospital of Chongqing Medical University for fever and seizures between October 1 and December 30, 2022. Based on the results of SARS-CoV-2 Reverse Transcription-Polymerase Chain Reaction(RT-PCR) at the time of admission, the patients were divided into a Coronavirus disease 2019(COVID-19) positive group and a COVID-19 negative group. Aside from that, we followed up COVID-19-positive patients for 3 months after their discharge from the hospital. The follow-up included monitoring for post-discharge seizures.

**Results:**

Compared with the COVID-19-negative group, the COVID-19-positive group had a higher proportion of seizure duration ≥ 15 min(18.7%VS5.1%;*P* = 0.001), seizure ≥ 2 time(54.4%VS41.0%; *P* = 0.024), status epilepticus(15.4%VS5.1%; *P* = 0.005), and Electroencephalogram (EEG) abnormalities(29.4%VS13.6%; *P* = 0.016). Among the 161 individuals under follow-up, 21 (13.0%)experienced a recurrence of seizures.

**Conclusions:**

Although the incidence of seizure duration ≥ 15 min, number of seizures ≥ 2 time, and status epilepticus was higher in the COVID-19-positive group, the majority of patients had a favorable prognosis. Nonetheless, patients with COVID-19 who present with seizures and persistent impaired consciousness need to be alerted to serious neurological disorders such as acute necrotizing encephalopathy. Owing to the consideration that some patients may experience a recurrence of seizures within a short period of time, it is paramount to provide guardians with education about the emergency management of seizures and to follow up with patients over time.

## Background

During the nascent phase of the Coronavirus disease 2019(COVID-19)pandemic, a majority of children infected with Severe Acute Respiratory Syndrome Coronavirus 2(SARS-CoV-2) exhibited fever and cough as the primary clinical features, with seizures being a comparatively rare occurrence [1]. Nevertheless, as the SARS-CoV-2 virus undergoes progressive mutations, the global prevalence of the Omicron variant has taken precedence [2]. Notably, recent investigations have shed light on an augmented frequency of seizure incidence in children when the omicron pandemic continues to ravage [3, 4]. Aside from that, a recent study reported severe neurological disease in several SARS-CoV-2 infected children, with fever and seizures as the main clinical manifestations [5]. Nonetheless, few studies so far have been found to probe into COVID-19 and seizures, and thereby even fewer related studies have been conducted to delve into the follow-up results. On that account, this study was predominantly intended to compare seizures in febrile children with and without SARS-CoV-2 infection and to conduct short-term follow-up of the SARS-CoV-2-infected patients.

## Methods

### Study population and definition

This retrospective study was conducted at the Children’s Hospital of Chongqing Medical University, one of the national pediatric medical centers in China. The inclusion criteria encompassed patients who were admitted for fever and seizures between October 1, 2022, and December 30, 2022. The exclusion criteria involved incomplete medical history records and previously existing neurological disorders diagnosed other than febrile convulsions. Patients were categorized into coronavirus disease 2019 (COVID-19)-positive and COVID-19-negative groups in accordance with the results of SARS-CoV-2 Reverse Transcription Polymerase Chain Reaction (RT-PCR) at the time of admission. Status epilepticus refers to a condition where the seizure lasts for longer than 30 min or when there is no recovery of consciousness between these two seizure episodes [6].

### Data collection

We collected the following information from the enrolled patients: age, gender, number of seizures, duration of the longest seizure, seizure types, accompanying symptoms, maximum temperature during the course of illness, results of the neurological exam, convulsion history, family convulsion history, blood test results, cerebrospinal fluid (CSF) test results, EEG(Electroencephalogram) results, and head CT (Computed Tomography) or MRI (Magnetic Resonance Imaging) imaging data. Aside from that, we followed up the patients in the COVID-19-positive group for a period of 3 months via outpatient clinic visits and telephone calls to follow up on the children’s seizures. Any differences in the data collection process were systematically and comprehensively analyzed through discussion.

### Statistical analysis

The Statistical Package for Social Sciences version 26.0 for Windows (SPSS Inc., Chicago, IL, USA) was used. Continuous variables were described as median and quartile intervals or mean and standard deviations, while categorical variables were described by numbers and percentages. Results were compared between groups by adopting a chi-square or Fisher exact test for categorical variables and an independent-sample T-test or Mann-Whitney U-test for continuous variables. *p-*value < 0.05 was considered statistically significant.

## Result

### Patient cohort

**Fig. 1:**
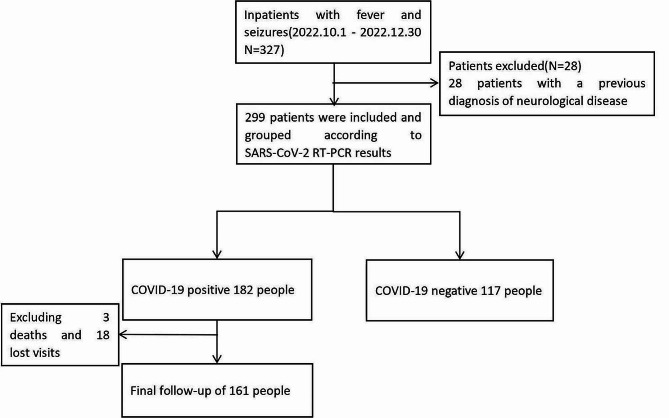
Flow chart of the study design

A total of 299 patients were enrolled in this study, with 182 in the COVID-19 positive group and 117 in the COVID-19 negative group. Among the COVID-19 positive group, 3 patients died, and we were unable to establish contact with 18 patients. As a result, our final follow-up cohort comprised 161 patients (Fig. 1).

### Comparison of data between COVID-19 positive and COVID-19 negative groups

In accordance with the results of SARS-CoV-2 reverse transcription-polymerase chain reaction (RT-PCR), the patients were divided into a COVID-19 positive group (*n* = 182) and a COVID-19 negative group (*n* = 117). Table 1 delineates the dissimilarities between these two cohorts. Our findings reveal a higher prevalence of seizure duration ≥ 15 min, seizure ≥ 2 times, status epilepticus, and EEG abnormalities in the COVID-19 positive group in comparison with the COVID-19 negative group.

**Table 1:** Comparison of data between COVID-19 positive and COVID-19 negative groups

Variable	COVID-19 positive group	COVID-19 negative group	*P*
N	182	117	
Male	107(58.8%)	67(57.3%)	0.794
Age (years)	2.3(1.3–3.8)	2.1(1.2–3.5)	0.239
≤1 years	25(13.7%)	17(14.5%)	0.847
1–5 years	130(71.4%)	85(72.6%)	0.819
≥5years	27(14.8%)	15(12.8%)	0.625
Time from fever onset to seizure			
<24 hours	163(89.6%)	101(86.3%)	0.396
≥24hours	19(10.4%)	16(13.7%)	0.396
Seizure types			
Generalized seizures	173(95.1%)	110(94.0%)	0.697
Partial seizures	9(4.9%)	7(6.0%)	0.697
Seizure duration ≥ 15min	34(18.7%)	6(5.1%)	0.001
Status epilepticus	28(15.4%)	6(5.1%)	0.006
History of convulsions	82(45.1%)	43(36.8%)	0.155
Family history of convulsions	42(23.1%)	29(24.8%)	0.736
Seizure ≥ 2 time	99(54.4%)	48(41.0%)	0.024
Peak body temperature, ℃	39.5(39.0–40.0)	39.5(39.0–40.0)	0.019
Abnormal EEG	20/68(29.4%)	12/88(13.6%)	0.016
Simultaneous phenomenon			
Pharyngodynia	10(5.5%)	6(5.1%)	0.891
Cough	79(43.4%)	51(43.5%)	0.975
Vomiting	52(28.6%)	20(17.1%)	0.023
Diarrhoea	9(4.9%)	8(6.8%)	0.490
Dizziness/Headache	8(4.4%)	2(1.7%)	0.352
Labiratory results			
WBC(*10^9^/L)	5.9(4.7–7.6)	8.7(6.3–12.8)	< 0.001
HB(g/dl)	12.1(11.6–12.7)	12.1(11.4–12.8)	0.295
PLT(*10^9^/L)	212(183–258)	239(205–309)	< 0.001
CRP(mg/L)	5.2(1.8–11.2)	9.0(2.8–30.7)	< 0.001

WBC: White blood cell count; HB: Hemoglobin; PLT: Blood platelets; CRP: C-reactive protein.EEG:Electroencephalogram

### COVID-19 relevant data for positive and negative groups

Among the cohort of 182 patients with fever and seizures tightly connected with COVID-19, 7 exhibited a poor prognosis, consisting of 3 fatalities (Table 2) and 4 individuals with residual partial cognitive or motor impairment. It’s particularly noteworthy that all 7 patients displayed impaired consciousness lasting for several hours, with 3 of them receiving a diagnosis of the exceedingly rare acute necrotizing encephalopathy (Table 3). Subsequently, 32 patients (17.6%) underwent routine cerebrospinal fluid analysis through lumbar puncture. This all-round analysis encompassed the assessment of appearance, pressure, protein, glucose, chloride ion levels, cell count, and cell classification. Within this subset, 28 patients exhibited normal cerebrospinal fluid results, 2 displayed elevated glucose levels, while 1 exhibited elevated protein levels, and another showed elevated levels of both leukocytes and protein in their cerebrospinal fluid.

**Table 2:** Data related to the three deceased patients

Variable	Patient 1	Patient 2	Patient 3
Age	3 years and 9 months	2 years and 2 months	2 years and 2months
Sex	Female	Female	Female
Main symptoms	Fever, seizures, impaired consciousness	Fever, seizures, impaired consciousness	Fever, seizures, impaired consciousness, double inspiration
Seizure forms	GTCS	GTCS	GTCS
Underlying Diseases	None	None	None
State of consciousness	Coma	Coma	Coma
With or without shock	Shock	Shock	Shock
EEG	None	None	Background diffuse low voltage changes
Hematology tests			
WBC(*10^9^/L)	5.7	3.89	9.72
CRP(mg/L)	7.2	0.5	10.5
PCT(ng/ml)	--	43.24	38.23
Treatment			
Ventilator	Invasive ventilator	Invasive ventilator	Invasive ventilator
Drugs	Methylprednisolone	Gammaglobulin	Methylprednisolone
	Gammaglobulin	Vasoactive drugs	Gammaglobulin
	Vasoactive drugs		
Head CT	None	Bilateral symmetric hypodense lesions in the thalami, accompanied by enlarged thalamic volume and similar low-density changes in the brainstem	The bilateral cerebellar hemispheres and thalamus exhibit symmetric patchy hypodense lesions. The density of the brainstem is reduced. In parts of the bilateral cerebral hemispheres, the grey-white matter differentiation is well-defined.

GTCS: generalized tonic-clonic seizure;WBC: White blood cell count;CRP: C-reactive protein;CT:Computed Tomography;EEG:Electroencephalogram;PCT:Procalcitonin

**Table 3:** Data from 2 patients diagnosed with ANE and 1 patient with highly suspected ANE

Variable	Patient 3	Patient 4	Patient 5
Sex	Female	Male	Male
Age	2 years and 2 months	11 months	2 months
Main symptoms	Fever, seizures, impaired consciousness, double inspiration	Fever, seizures, impaired consciousness	Fever, seizures, impaired consciousness
Seizure forms	GTCS	GTCS	GTCS
Primary Diagnosis	ANE	ANE	Highly suspicious of ANE
Cerebrospinal fluid			
WBC(*10^6^/L)	None	1	19
GLU(mmol/L)	None	2.85	2.34
PRO(g/L)	None	4.04	3.01
EBV(PCR)	None	Negative	Negative
HSV(PCR)	None	Negative	Negative
CMV(PCR)	None	Negative	Negative
Cultivation	None	Negative	Negative
mNGS	None	Negative	None
Hematology tests			
WBC(*10^9^/L)	9.72	9.06	5.94
CRP(mg/L)	10.51	17.66	0.71
PCT(ng/ml)	38.23	> 100	0.17
ALT(U/L)	222	1307	32
AST(U/L)	241	1970	48
SF(ng/ml)	907.4	None	None
IL-6(pg/ml)	None	109.1	None
CT or MRI of the head	The bilateral cerebellar hemispheres and thalamus exhibit symmetric patchy hypodense lesions. The density of the brainstem is reduced. In parts of the bilateral cerebral hemispheres, the grey-white matter differentiation is well-defined	Bilateral thalami, basal ganglia, brainstem, and cerebellum show symmetric large areas of hypodensity with partial swelling	The bilateral cerebral hemispheres demonstrate widespread hyperintense signals on both T1 and T2 weighted images. Similar signal abnormalities are observed in the bilateral thalamus, internal capsule, and brainstem
Main treatment ventilator			
Drugs	Invasive ventilator	Invasive ventilator	None
	Methylprednisolone	Methylprednisolone	Methylprednisolone
	Gammaglobulin	Gammaglobulin	Gammaglobulin

ANE:Acute necrotizing encephalopathies;WBC: White blood cell count;CRP: C-reactive protein;PCT:Procalcitonin;

PRO:Protein;EBV:Epstein-Barr virus;HSV:Herpes simplex virus;CMV:Cytomegalovirus;mNGS:metagenomic next-generation sequencing;ALT:Alanine aminotransferase;AST:Aspartate aminotransferase;SF:Serum ferritin;IL-6:Interleukin-6

Among the 117 patients in the COVID-19-negative group, 98 were diagnosed with febrile convulsions, 4 were diagnosed with CNS infections, and the remaining 15 could not be definitively diagnosed at this time as a consequence of their refusal to be examined. In the COVID-19-negative group, 20 patients underwent lumbar puncture cerebrospinal fluid (CSF) examination. Among them, one patient exhibited augmented cerebrospinal fluid proteins and leukocytes, while the remaining 19 patients displayed normal CSF results.

### Follow-up results

Among the 161 individuals under follow-up, 21 (13.0%) experienced a recurrence of seizures—specifically, 3 presented non-febrile seizures, while 18 exhibited febrile seizures.

## Discussion

As illustrated by relevant study, COVID-19 variant Omicron infection was accompanied by more severe seizures, as evidenced by a higher proportion of seizure duration ≥ 15 min, seizure ≥ 2 time, persistent status epilepticus, which has been similarity proven by a relevant study [4]. As persuasively demonstrated by previous studies, fevers may trigger febrile seizures via the inflammatory pathway, but different pathogens do not result in the same inflammatory mediators, combined with studies suggesting that inflammatory mediators may be tied up with status epilepticus [7]. As a consequence, we hypothesize that this more severe seizure may be tightly correlated with the inflammatory response after COVID-19 infection.

Similar to previous coronaviruses, SARS-CoV-2 can easily enter the nervous system and give rise to neurological symptoms. The virus may enter the central nervous system through the olfactory pathway and angiotensin receptor-2 [8], bringing about astrocytosis and microglial activation. This phenomenon, in turn, triggers the release of pro-inflammatory cytokines (TNF-α, IL-6, IL-1B), which conducts a pivotal role in the seizure mechanism. Nevertheless, pro-inflammatory factors in the central nervous system are not derived solely from astrocytes. To be special, when the blood-brain barrier is damaged or underdeveloped, it becomes another route for pro-inflammatory factors to enter the brain. This may also throw light upon a fundamental fact that seizures occur even in the absence of infection of the central system. Aside from that, high fever after new crown infection is also an agent of seizures. As clearly revealed by relevant studies, hyperthermia not only has a disadvantageous effect on neurons, but also induces the release of inflammatory mediators and heightens the permeability of the blood-brain barrier [9–11]. As exhibited in our research, the peak fever of 39.5 ± 0.7 °C in the COVID-19-positive group was higher than the average peak fever of 38.9 ± 0.6 °C in Omicron-infected children [12], which forcefully verifiss an essential fact that hyperthermia after COVID-19 infection is a factor of convulsive episodes.

Another potential factor of seizures in COVID-19 patients is direct damage to the central nervous system by the virus. Nevertheless, a recent systematic review of cerebrospinal fluid testing in seizure patients with COVID-19 found little evidence to support this theory, as the virus was detected in only a small percentage of patients [13]. As illustrated in our study, 32 (17.6%) patients in the COVID-19 positive group completed routine cerebrospinal fluid (CSF) examination. It’s tremendously noteworthy that only four of these patients had abnormal cerebrospinal fluid. Under this circumstance, such results do not support seizures resulted from viral encephalitis. Nonetheless, since we have not performed COVID-19 PCR tests on cerebrospinal fluid, it cannot be ruled out that COVID-19 invades the brain and causes seizures. As a result, in the future, it is crucial to conduct a more comprehensive neurological evaluation and test on COVID-19 in cerebrospinal fluid for patients suspected of having central nervous system invasion by COVID-19, instead of relying upon nothing more than routine CSF tests.

Despite the fact that cerebrospinal fluid examination does not support seizures owing to direct invasion of the central nervous system by COVID-19, we must emphasize the possibility of severe neurological disorders secondary to COVID-19 infection. In the present study, 9 patients with COVID-19 infection developed severe neurological manifestations in the form of impaired consciousness lasting several hours and recurrent seizures or persistent status epilepticus. Among them, 3 patients died, and 2 of them shifted quickly from fever to death within just 2 days. Owing to the seriousness of these 2 patients, lumbar puncture cerebrospinal fluid examination and neurological MRI auxiliary examination could not be completed, and their family members refused to authorize an autopsy. For this reason, the final etiology could not be determined at that time. Although it remains unclear whether COVID-19 directly resulted in these deaths, these cases should be highlighted to capture the attention of physicians and researchers, so as to suggest more effective treatment measures.

Apart from that, 2 patients were definitively diagnosed with Acute Necrotizing Encephalopathy (ANE), a rare and severe encephalopathy usually stemmed from a viral infection. The clinical presentation, imaging, and laboratory findings of these 2 patients met the diagnostic criteria for ANE, namely seizures, impaired consciousness, and imaging manifestations of symmetrical thalamic and multisite lesions. It is worth discussing the other patients in this study. These patients presented with seizures and impaired consciousness, and MRI of the head suggested abnormal signal shadows in the bilateral thalamus, internal capsule, and cerebral hemispheres. Such clinical presentation and imaging findings led the physician to consider necrotizing encephalopathy, but cerebrospinal fluid examination in this patient suggested slightly elevated white blood cells, which was not in line with the previous diagnostic criteria for ANE. Nonetheless, a recent study reported a case of COVID-19 infection with typical clinical and imaging manifestations of ANE, but the patient’s cerebrospinal fluid examination suggested leukocytosis, and the patient was finally diagnosed with ANE anyway after pathological examination of the thalamus and metagenomic Next-Generation Sequencing (mNGS) of the cerebrospinal fluid [14]. The prognosis of all three patients considered for ANE in this study was not so satisfactory as expected. Among them, one patient died, one patient left with motor dysfunction, and one patient whose guardian ultimately chose to abandon treatment owing to slim hope of cure. Consequently, in patients who develop seizures after COVID-19 infection, physicians need to be alert to the possibility of secondary necrotizing encephalopathy in the children, especially those who develop persistent impairment of consciousness.

Nonetheless, notwithstanding the fact that we have made major achievements, there were still several limitations in our study. First and foremost, this is a single-center study, but given that our hospital is the largest children’s hospital in southwest China, our patients are somewhat regionally representative. Apart from that, to ensure the reliability and integrity of our data, we only included inpatients and excluded outpatients. Last but not least, on account of various factors, some patients have not completed relevant examinations, which may give rise to deviations in the results, but the clinical data on seizures are far beyond our expectation.

## Conclusions

Although a higher proportion of the COVID-19 positive group experienced seizure duration ≥ 15 min, seizure ≥ 2 time, status epilepticus, the vast majority of patients present a desirable prognosis. Nevertheless, in patients with persistent impairment of consciousness after COVID-19 infection, the prognosis may be dissatisfactory and requires vigilance for secondary serious neurological disease. What’s more, some patients may experience a recurrence of seizures within a short period of time. Based on these characteristics, it is fairly essential for medical personnel to actively inform patients’ family members of the risk of recurrent seizures, provide sufficient education on emergency seizure management, and conduct regular follow-up visits for this patient population so as to ensure patients’ safety and well-being.

## Data Availability

The datasets used and/or analysed during the current study are available from the corresponding author on reasonable request.

## Abbreviations

SARS-CoV-2: Severe acute respiratory syndrome coronavirus 2
COVID-19: Coronavirus disease 2019
FS: Febrile seizures
RT-PCR: reverse transcription-polymerase chain reaction
EEG: Electroencephalogram
CSF: cerebrospinal fluid
CT: Computed Tomography
MRI: Magnetic Resonance Imaging
WBC: White blood cell count
HB: Hemoglobin
PLT: Blood platelets
CRP: C-reactive protein
NS: Not significant in terms of statistics
ANE: Acute necrotizing encephalopathies
PCT: Procalcitonin
PRO: Protein
EBV: Epstein-Barr virus
HSV: Herpes simplex virus
CMV: Cytomegalovirus
mNGS: metagenomic next-generation sequencing
ALT: Alanine aminotransferase
AST: Aspartate aminotransferase
SF: Serum ferritin
IL-6: Interleukin-6

## References

[1] Dong Y, Mo X, Hu Y, et al. Epidemiology of COVID-19 among children in China[J]. Pediatrics. 2020;145(6). 10.1542/peds.2020-0702 10.1542/peds.2020-0702 32179660

[2] Tian D, Sun Y, Xu H (2022). The emergence and epidemic characteristics of the highly mutated SARS-CoV-2 Omicron variant[J]. J Med Virol.

[3] Pascarella A, Maglione M, Lenta S, et al. Seizures in children with SARS-CoV-2 infection: epidemiological, clinical and neurophysiological Characterization[J]. Child (Basel). 2022;9(12). 10.3390/children9121923 10.3390/children9121923 PMC9777450 36553366

[4] Joung J, Yang H, Choi YJ (2023). The impact of Omicron Wave on Pediatric Febrile Seizure[J]. J Korean Med Sci.

[5] Chen CS, Chang CN, Hu CF (2022). Critical pediatric neurological illness associated with COVID-19 (Omicron BA.2.3.7 variant) infection in Taiwan: immunological assessment and viral genome analysis in tertiary medical center[J]. Int J Infect Dis.

[6] Tintinalli J, Ma OJ, Yealy D, Meckler G, Stapczynski J, Cline D (2019). Tintinalli’s Emergency Medicine: a Comprehensive Study Guide.

[7] Dono F, Nucera B, Lanzone J (2021). Status epilepticus and COVID-19: a systematic review[J]. Epilepsy Behav.

[8] Steardo L, Steardo L, Zorec R (2020). Neuroinfection may contribute to pathophysiology and clinical manifestations of COVID-19[J]. Acta Physiol (Oxf).

[9] Nikbakht F, Mohammadkhanizadeh A, Mohammadi E (2020). How does the COVID-19 cause seizure and epilepsy in patients? The potential mechanisms[J]. Mult Scler Relat Disord.

[10] Kiyatkin EA, Sharma HS (2009). Permeability of the blood-brain barrier depends on brain temperature[J]. Neuroscience.

[11] Reid AY, Galic MA, Teskey GC (2009). Febrile seizures: current views and investigations[J]. Can J Neurol Sci.

[12] Wang X, Chang H, Tian H (2022). Epidemiological and clinical features of SARS-CoV-2 infection in children during the outbreak of Omicron variant in Shanghai, March 7–31, 2022[J]. Influenza Other Respir Viruses.

[13] Carroll E, Melmed KR, Frontera J (2021). Cerebrospinal fluid findings in patients with seizure in the setting of COVID-19: a review of the literature[J]. Seizure.

[14] Rettenmaier LA, Abdel-Wahed L, Abdelmotilib H (2022). COVID-19-associated necrotizing encephalopathy presenting without active respiratory symptoms: a case report with histopathology[J]. J Neurovirol.

